# Sociodemographic profile and prescribing pattern of antipsychotic medication in patients with Schizophrenia

**DOI:** 10.1192/j.eurpsy.2024.514

**Published:** 2024-08-27

**Authors:** A. Palushaj, V. Kola

**Affiliations:** ^1^Neuroscience, “Ihsan Çabej” Hospital, Lushnje; ^2^Neuroscience, University Hospital “ Mother Teresa”, Tirane, Albania

## Abstract

**Introduction:**

Schizophrenia is a complex psychiatric disorder that changes the patient’s life by influencing how they think, behave, express emotions, percept reality and their interpersonal relationships.

**Objectives:**

The aim of this study was to evaluate sociodemographic and therapeutic factors that act as risk and protective factors in the clinical outcomes of patients diagnosed with schizophrenia.

**Methods:**

This was an observational retrospective study including patients diagnosed with schizophrenia, treated at the “Xhavit Gjata” Psychiatric Hospital, Tirane, Albania, who were discharged between May 1- October 30, 2022. The follow-up period was six months. Data on further hospitalizations during the follow-up were obtained from the Department of Statistics, QSUT, and confirmed by family members for hospitalizations in other psychiatric hospitals in the country. Univariate and multivariate analyses were conducted to identify potential factors associated with emergency room stays, length of stay, and time until the next admission.

**Results:**

A total of 158 patients were included in the study, 63 women and 95 men (p=0.03). The average age of the patients was 42.9 years, with women averaging 45.3 years and men 40.6 years (p=0.01). 43.7% of them had elementary education. The average age of disorder onset was 24.7 years. Haloperidol was the ambulatory therapy used in 54.3% of patients, while atypical antipsychotics were used in 75.1% of patients. The most commonly used atypical antipsychotic was Risperidone in 34.1% of patients, followed by Olanzapine in 17% of cases. Depo antipsychotics were used in 35.1% of patients. Clozapine was administered to 29.3% of patients, where 12.8% for the first time. 54.2% of patients starting Clozapine for the first time had three or more admissions. Clozapine was more frequently used in men, showing a significant difference from women (p=0.05). In 44.7% of cases, monotherapy was prescribed. The average hospital stay was 21.9 days, ranging from 2-68 days. Living with a family member, male gender, and being “married” helped reduce the length of hospital stay. In the 6-month follow-up period, 31.4% were re-hospitalized. Significant factors affecting the reduction of time spent outside the hospital until the next hospitalization were social problems, the number of previous hospitalizations, civil status “not married,” living arrangements, negative symptoms, and alcohol use (nearly significant). Protective factors included Clozapine, which reduced the prevalence of hospitalization by 57% compared to patients not taking it. Additionally, the use of Clozapine and Haloperidol increased the time spent outside the hospital.

**Conclusions:**

Social and family support, positive compliance, and antipsychotic therapy such as Clozapine serve as protective factors for patients diagnosed with schizophrenia.

**Disclosure of Interest:**

None Declared

